# An Ontology-Based Framework for Psychological Monitoring in Education During the COVID-19 Pandemic

**DOI:** 10.3389/fpsyg.2021.673586

**Published:** 2021-07-22

**Authors:** Alia El Bolock, Slim Abdennadher, Cornelia Herbert

**Affiliations:** ^1^Department of Applied Emotion and Motivation Psychology, Institute of Psychology and Education, Ulm University, Ulm, Germany; ^2^Department of Computer Science, Faculty of Media Engineering and Technology, German University in Cairo, Cairo, Egypt

**Keywords:** COVID 19, psychological ontologies, mental health, character computing, education, emotion, personality

## Abstract

**Background:**

Especially in the current crisis of the COVID-19 pandemic and the lockdown it entailed, technology became crucial. Machines need to be able to interpret and represent human behavior, to improve human interaction with technology. This holds for all domains but even more so for the domain of student behavior in relation to education and psychological well-being.

**Methods:**

This work presents the theoretical framework of a psychologically driven computing ontology, CCOnto, describing situation-based human behavior in relation to psychological states and traits. In this manuscript, we use and apply CCOnto as a theoretical and formal description system to categorize psychological factors that influence student behavior during the COVID-19 situation. By doing so, we show the added value of ontologies, i.e., their ability to automatically organize information from unstructured human data by identifying and categorizing relevant psychological concepts.

**Results:**

The already existing CCOnto was modified to automatically categorize university students’ state and trait markers related to different aspects of student behavior, including learning, worrying, health, and socially based on psychological theorizing and psychological data conceptualization.

**Discussion:**

The paper discusses the potential advantages of using ontologies for describing and modeling psychological research questions. The handling of dataset completion, unification, and its explanation by means of Artificial Intelligence and Machine Learning models is also discussed.

## Introduction

The traits and predispositions of an individual are central to their self-regulation capabilities, and subsequently, their emotional state change in response to a specific situation (Gramzow et al., 2004; McCrae and Löckenhoff, 2010). One such situation is the recent outbreak of the COVID-19 virus and the lifestyle changes it entailed, including the lockdown most countries suffered from in the first wave to the physical and mental health concerns. One crucially affected sector is education, as students, teachers, and families had to adjust to an unprecedented change in the format of the educational system (d’Orville, 2020). The mental well-being of all involved parties requires attention and maintenance, as they are central to their performance quality. Learners, i.e., students, are the most malleable of the involved parties. It is essential to focus on them specifically and how educators and family members can gain more awareness about their emotional states and help them during crises (Chandra, 2020; Raaper and Brown, 2020).

Personality-specific learner styles (Kamarulzaman, 2012; Siddiquei and Khalid, 2018), (personality-specific) emotional responses to crises (Roth, 2018; Restubog et al., 2020), and the effect of emotional states on educational performance (Johnson et al., 2017; Hase et al., 2019; Akpur, 2020) have been extensively investigated in the literature. However, a combination of all factors still has fewer numbers of published insights. Thus, there is a need and pandemic-lead urgency for a joint framework that enables the description, analysis, evaluation of the factors related to student’s mental well-being within the education framework.

There are many existing computational frameworks aiming at representing and predicting human factors [see Rodríguez et al. (2014); Blanch et al. (2017); Abaalkhail et al. (2018); and Norris et al. (2019)]. Notably, in Larsen and Hastings (2018), ontologies have been utilized to map psychological concepts between two existing ontologies for affective processes and psychiatric diseases. The proposed approach can already be considered a categorization task where the entities of one ontology are categorized based on the others. Existing approaches are (1) uni-modal in terms of underlying theories representing the different factors, (2) do not include all factors relevant to indicating behavior in a learning context during the pandemic, and (3) are often application-specific.

This paper presents a more self-contained concept of ontology-based data organization and creation by basing it on a generic adaptable ontology of human behavior and its factors. It first starts with a general explanation of ontologies, describes already existing ontologies and their application in psychology and life science, including education. Then, we introduce potential extensions of an existing psychologically driven ontology CCOnto [see El Bolock et al. (2021) and El Bolock et al. (2020c)]. In this paper, we show how CCOnto was modified and applied as an automated formal knowledge system to describe self-reported changes in student behavior associated with the current COVID-19 pandemic based on psychological theorizing and psychological data conceptualization as described in Herbert et al., 2021a.

## Background: Ontologies

With the emergence of vast amounts of data from different contexts, recorded in different formats and modes, the focus in scientific research has shifted from focusing on how to collect data to how to organize and extract meaningful information from existing data created by humans. The need for interdisciplinary research, cross-disciplinary understanding, and the shareable integration of domain knowledge, including psychological theories and concepts, has become apparent. Ontologies are one often used approach addressing the aforementioned problems.

Ontologies have been defined and used by different with various meanings but all for similar purposes [for a recent terminological definition of the term, see Stancin et al. (2020)]. At its essence, an ontology can be regarded as a taxonomy organizing concepts in a specific domain or collection of domains and relations between them (denoted T-Box). For example, we can define the relation “HasTrait” between two conceptual entities, “human” and “trait.” Additionally, we can define instances (specific individuals that belong to the defined entities), e.g., the human “Annie” who has the “anxiety” trait (denoted A-Box). Finally, we can define constraints or restrictions on the different entities and individuals, like saying that a “human” is a subset of “Living being” (denoted R-Box). The most significant benefit of ontologies is that, aside from being easily interpretable by human users, they are automatically processable by algorithms and automatic reasoners. This enables implicitly inferring information included in the represented knowledge or new information.

Ontologies are efficient means for integrating, structuring, and sharing knowledge because the common good practice of ontology engineering hinges on reusing and integrating existing ontologies (Stancin et al., 2020). This means that whenever a new ontology representing a knowledge domain is needed, the ontology engineers need to check all existing ontologies that contain entities needed for the new ontology or overlap with parts of the ontology. These ontologies and their overlapping terms need to be reused within the new ontology instead of redefining them as separate entities. This results in substantial interconnected networks of terms that are all somehow linked to each other through different ontologies, enabling drawing new connections and having an overview of the different knowledge parts and how they fit within a bigger picture.

One star application field utilizing ontologies is bioinformatics and health care. The most prominent example is the Gene Ontology (GO) (Ashburner et al., 2000; Consortium, 2019) which is being maintained, updated, and used since 1998 and is referenced in thousands of scientific publications over the years. Numerous similar ontologies are being used and are collected within the different repositories and following predefined standards like the Open Biological and Biomedical Ontology Foundry (OBO).

Ontologies have been successfully applied in different domains either as a stand-alone standardization effort or as part of semantic web applications, such as education [e.g., Stancin et al. (2020)], behavior [e.g., Blanch et al. (2017)], behavior change [e.g., Norris et al. (2019)], affective research [e.g., Abaalkhail et al. (2018)], and healthcare [e.g., Dimitrieski et al. (2016)]. Ontologies provide structured annotations to diverse and seemingly non-compatible data and enable the analysis of raw data in an automated way while still being flexible to accommodate different theories and compare different findings. The latter is realized implicitly due to ontologies’ structure, as one can define all the possibly existing entities and relations within a specific domain of knowledge (T-Box). Still, only input individuals to a subset of those (A-Box), thus temporarily excluding the unused entities if needed. The R-Box constraints can also be manipulated depending on the researcher’s current hypothesis or used to compare the alignment of input data to the hypothesized constraints.

### Ontologies in Education

An overview of existing ontologies related to education can be found in Stancin et al. (2020). Ontologies about education can be categorized into four main categories: curriculum modeling and management, describing learning domains, describing e-learning services, and describing learning data. Some ontologies cover multiple categories at the same time. Of particular interest for this paper are ontologies belonging to the final category. Ontologies describing learning data mainly focus on modeling learners and their related data. LifeOn is a “ubiquitous lifelong learner model ontology” (with a highlight on learner personality) for adaptive learning systems (Nurjanah, 2018). LifeOn represents static (special needs, preferences, profile) and dynamic (learning history and study plan) attributes of learners with academic and non-academic scopes. In Akharraz et al. (2018), an ontology for reasoning about learners and their needs is presented. The goal of the ontology is to help learners realize intended learning goals by allowing exchanging the learners’ profiles between the components of the e-learning system. Han (2018) presents an interdisciplinary ontology-based model aiming to improve students’ cognitive abilities. The model serves as a guide for building teaching plans by helping college educators understand the learning situation of the individual students. An ontology for modeling learning preferences and predicting learning styles is presented in Rami et al. (2018). Grivokostopoulou et al. (2019) present an ontology used to analyze student’s learning and performance within the context of a tutoring system for Artificial Intelligence. The system aims at supporting both students and teachers. Zhao and Guo (2019) present how big data can be leveraged to provide personalized distance learning while avoiding information resource overload. They propose an ontology to model user interests based on user information and preferences. The learning management system presented in Bouquet and Molinari (2016) represents heterogeneous information through semantic knowledge bases to ensure expressivity and reusability. In Rani et al. (2016), an ontology is used to improve data representation and learner classification within a learning management system. However, learner profiling is only done based on demographic information. A multimodal ontology-based school care system targeted toward students with special educational needs is presented in Hafidh et al. (2019). The presented application combines multiple factors such are education, health, and social interaction. The e-learning framework presented in Sarwar et al. (2019) aims at profiling and categorizing learners to provide content recommendations and adaptations. However, the ontology is only utilized to represent the learning content and not for the learner profiling and recommendation process. In Saleena and Srivatsa (2015) and Kaur et al. (2015), e-learning systems integrating multiple ontologies for modeling learner profiles, with the former being more oriented toward learner evaluation and the latter focusing on the learner’s personality.

### Ontologies Related to Human Behavior

Several approaches have been proposed for using ontologies when representing and modeling the complex interactions between human behavior, personality, and emotions. The Mental Functioning Ontology (MF) is a general ontology of aspect of mental functioning Hastings et al. (2012). It represents mental processes (including cognition and general traits). The ontology complies and is aligned with the Ontology for General Medical Science (OGMS) (Ceusters and Smith, 2015) and the Basic Formal Ontology (BFO) (Arp et al., 2015) which represents concepts as continuant (e.g., humans, cognitive representations, and dispositions) or occurrent (e.g., processes like behavior) entities. MF is also partially aligned with the Cognitive Paradigm Ontology (Turner and Laird, 2012) and the Cognitive Atlas (Poldrack et al., 2011). The ontology has two extension modules the Mental Disease Ontology (MFOMD) (Ceusters and Smith, 2010; Hastings et al., 2012) and the Emotion Ontology (MFOEM) (Hastings et al., 2011b, a). The former represents mental disorders in alignment with the Disease Ontology. The latter represents emotions and all related affective phenomena. MFOEM models bearers of affective phenomena, different emotion types, moods, as well as their different building blocks, dimensions, physiological and bodily representation markers (e.g., facial expressions). It also represents the relation between affective phenomena and human behavior. MF and its modules support alignment with self-reporting or articulation of emotional states and responses (Hastings et al., 2014; Larsen and Hastings, 2018). EmOCA, an emotion ontology, can be used to reason about philia and phobia based on emotion expression in a context-aware manner (Berthelon and Sander, 2013). EmotionsOnto is another emotions ontology for developing affective applications and detecting emotions (López Gil et al., 2014). In García-Vélez et al. (2018), an ontology of psychological user profiles (mainly personality traits and facets) is presented. A web application for detecting personality using linguistic feature analysis based on ontologies of personality and other techniques is presented in Sewwandi et al. (2017). An ontology for insider threat risk detection and mitigation through individual (personality, affect, ideology, and other similar attributes) and organizational socio-technical factors is presented in Greitzer et al. (2016). The HeLiS ontology (Bailoni et al., 2016; Dragoni et al., 2018) models the concepts representing the food and physical activity domains. Through modeling detailed food properties and physical activity properties, HeLiS supports the construction of intelligent interfaces for domain experts to support a healthy lifestyle and is extended to representing behavior change in this aspect (Dragoni and Tamma, 2019). Another ontology for representing the psychological barriers preventing behavior change and thus enabling overcoming them is presented in Alfaifi et al. (2018). The ontology does that from the perspective of patient behavior in relation to the necessary behavior change interventions for type 2 diabetes.

The above-discussed ontologies each cover one specific domain or factor of human experience and behavior. Crucially, the represented domains and human factors are based on one specific defining theory, e.g., the two-factor theory of emotions or appraisal theory of emotions, in Berthelon and Sander (2013) and Hastings et al. (2014), respectively. However, human behavior and the psychological factors that influence it are much more complex and not just dependent on one single factor. Therefore, ontologies need to have the flexibility to include and combine different psychologically relevant attributes to construe a formal description of the many factors and their inter-relationship and their relevance for behavior.

In line with this, an integrated ontology of human character and its interaction with behavior and situation is necessary to enable the automated description of human behavior in a situation-sensitive manner, considering overt and covert human factors [for an overview, see Herbert et al. (2020), Herbert (2020)].

To accomplish this aim, computer scientists and psychologists need to work together to build a psychology-driven ontology.

### CConto as a Psychologically Driven Ontology for Describing Human Behavior Based on Character Traits and States

**FIGURE 1:**
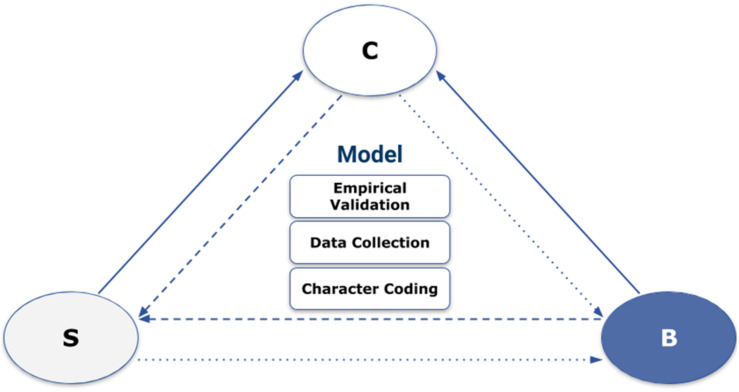
The Character – Behavior – Situation (CBS) triad of Character Computing and the building blocks of achieving a psychologically driven computation model, namely empirical validation, data collection, and ontology-based character coding.

Character Computing is a psychologically driven framework that considers an individual’s state and trait markers, represented as character [for an overview, see El Bolock (2020), Herbert (2020), and Herbert et al. (2020)] to predict human behavior in a specific situation [for more research, see El Bolock et al. (2020a)]. The interaction between these components is referred to as the Character – Behavior – Situation (CBS) triad [see El Bolock (2020); Herbert (2020), and Figure 1). As outlined in Herbert (2020), multiple psychological theories define human behavior and its underlying processes; and affecting factors. The inter-disciplinary field of Character Computing aims to validate, enrich, and possibly update our current understanding of human behavior in various contexts, e.g., education, well-being, health, and human computer interaction. This is done through a joint approach focused on experimental validation and computational, heuristic modeling, as shown in Figure 1 and outlined in Herbert et al. (2020). To provide a holistic definition of human behavior, we need to include overt and covert human factors, i.e., cognitive, affective, and motivational state, personality traits, subjective experiences, and socio-cultural embedding, e.g., Herbert et al. (2020). Following an interactionist psychological approach, human factors interacting with the situation to produce variability in behavior are defined as character within Character Computing. This approach accounts for the interactions between behavior, cognition, and emotions, aligning Character Computing with Artificial Intelligence and agent-based, embodied approaches. This differentiates the use of the term character from its common use in psychology, representing biologically based individual differences (temperament) or latent person variables defined by personality traits (Herbert, 2020). As behavior reactions feedback to an individual’s current state, the behavior needs to be dynamically modeled, as enabled by the CBS triad. A psychologically driven computation approach to Character Computing enables the extraction of behavior patterns and indicative features from data to predict certain human behaviors. By defining human behavior as an artifact sensitive to intra- and inter-individual differences (including language and culture) and resulting from the interactions between character and situation, we enable developing human-centered technology solutions.

Computational ontologies are semantic frameworks that structure and represent knowledge of a specific domain as a formal model. Similar to ontologies in Psychology, computational ontology models consist of domain-relevant concepts, the relationships between them, and the conditions constraining them. Computational ontologies have the advantage of providing a unified representation of a specific domain of knowledge that is shareable among humans and computer systems alike. Automated logic-based reasoning enables the inference of new or implicit knowledge from the explicitly defined domain. Ontologies have successfully been used to model mental processing, human emotions, behavior, and behavior change. Ontologies have also been often used for representing academic knowledge and as the underlying knowledge bases of education applications.

### Aim of the Present Study

Building on existing ventures, including those mentioned above [e.g., Larsen and Hastings (2018), Maimone et al. (2018), Dragoni et al. (2020), and Hastings et al. (2020)], in this manuscript, we present how an ontology is used to identify and structure university student behavior data during the first COVID-19 lockdown (Herbert et al., 2021a).

As described in more detail in the section ‘‘Methods,’’ the already developed ontology, CCOnto^1^, represents knowledge about behavior, situation, and character [see El Bolock et al. (2021, 2020c)]. It is, therefore, an ideal framework that can serve as a common ground for psychoeducational research and provide further insights into the involved concepts to be used as a basis for psychology- and technology-based solutions. A set of existing tools can be used to enable psychologists to browse the relevant ontology parts (Lohmann et al., 2016; Dudáš et al., 2018; Ivanova et al., 2019; El Bolock et al., 2020e; Florrence, 2021) and to apply rules to the knowledge represented by the ontology (Heyvaert et al., 2018; Coelho et al., 2019; El Bolock et al., 2020d; Pittl and Fill, 2020).

The data is modeled to categorize the essential variables defining and affecting the emotional well-being of learners. This enables pinpointing underlying causes for individual students’ different emotional states, predicting other students’ emotional responses, and conveying these predictions and understanding to caregivers (educators and family members). The ontology can be coupled with machine learning to explain black-box output predictions of machine learning models.

## Methods

### The Character Computing Ontology

CCOnto includes all relevant aspects of character- and behavior-based phenomena in an embodied contextual sense represented by the CBS Triad [first introduced in El Bolock (2020)]. This includes trait markers (e.g., personality and socio-cultural embeddings) and state markers (e.g., affect and well-being) that form the individual character, the models representing them, and the different theories on how they impact behavior in specific situations. The recommended best practice ontology development features include modularity, ontology reuse, continuous collaboration, and agile development based on rapid iterative prototyping (Kotis et al., 2020).

**FIGURE 2:**
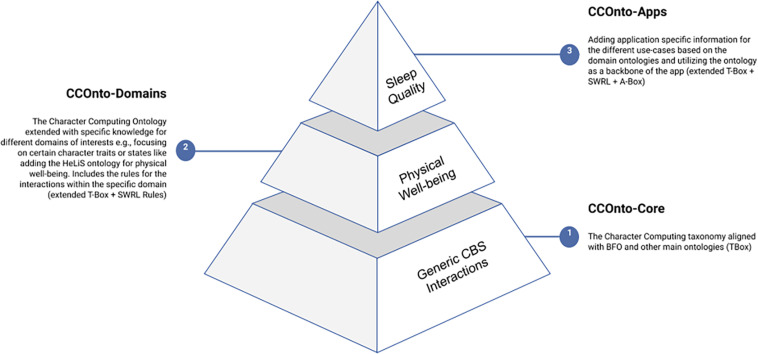
The three interconnected artifacts of the Character Computing Ontology.

CCOnto is modular, allowing the extension of specific concepts depending on the different use-cases, as shown in Figure 2. Our process of providing a model for the Character Computing framework is thus composed of three interconnected parts. The first, taxonomization, aims to collect all involved concepts and processes into a unified vocabulary while aligning them with existing taxonomies and categorizing ontologies. The second, contextualization, aims to extend the taxonomy regarding different contexts (domains) to represent behavior from an embodied perspective as rule-based functions of interactions of character states and traits with situation determinants. The third application aims to ground behavior, trait, state, and situation variables concerning domain-specific applications to harness the extracted contextual behavior concepts, as outlined in El Bolock et al. (2020b). CCOnto builds on existing efforts of ontologies representing mental functioning, emotions, context-awareness, health, and other related concepts. Specifically, the core part of CCOnto, CCOnto-Core, draws on the Mental Functioning Ontology (Smith and Ceusters, 2010), two of its branches: the Emotions Ontology (Hastings et al., 2014, 2011a,2011b), the Mental Disease Ontology (Hastings et al., 2012 and Larsen and Hastings, 2018), and the Emotions for Context Awareness Ontology (Berthelon and Sander, 2013). The relevant concepts are updated to fit CCOnto and combined with our own concepts. CCOnto-Domain integrates (one or more) existing domain ontologies covering the needed extension domains of interest, e.g., Dragoni et al. (2018). Finally, CCOnto-Apps can build domain-specific applications that use CCOnto-Domain for reasoning about concepts within the ontology based on specific use-cases. For example, we would use the proposed approach to assist educators by indicating the anxiety threat levels of different students based on their behavior for early interference.

The ontology is developed following the agile METHONTOLOGY ontology development methodology (Fernández-López et al., 1997). The methodology consists of five iterative steps whose intermediate output is validated and evaluated by the knowledge engineers and the domain experts. The five steps are (1) specification of the ontology purpose and domain, (2) conceptualization of the domain knowledge and reusing existing ontologies, (3) formalization of the conceptual model, (4) implementation of the formal model into a machine-readable format, and (5) maintaining, documenting and publishing the ontology. The development steps are described in detail in El Bolock et al. (2021). The ontology is written in the Web Ontology Language OWL 2.0 (World Wide Web Consortium, 2012) and implemented using the Protégé tool 5.2.1 (Musen, 2015), enabling rapid prototyping.

**FIGURE 3:**
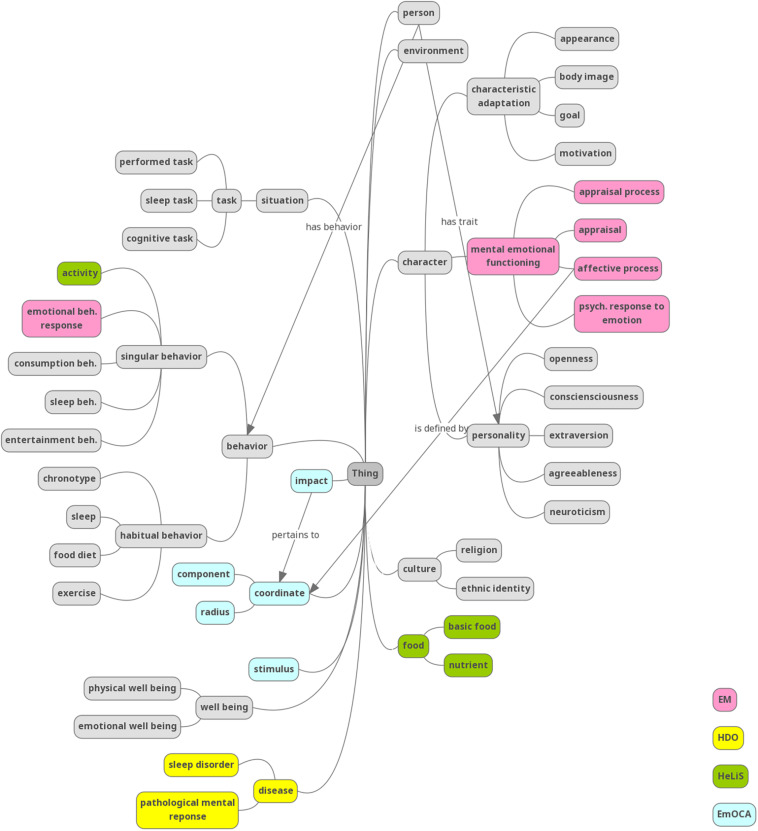
An overview of the hierarchy of CCOnto (in gray) and the main integrated concepts. Unlabeled arrows represent a relations.

A schematic overview of the main entities and relations included in CCOnto is shown in Figure 3, where the entities belonging to different existing ontologies are denoted in unique colors.

Table 1 gives an overview of the main class hierarchy. Only a representative sample of the classes and subclasses relevant to the scope of this paper is included. For example, the focus is on behavior and behavior change during the pandemic. The character, behavior, and situation concepts are formalized as a hierarchy of classes related through parent-child relationships.

**TABLE 1:** The abstracted hierarchy of the main upper-level classes of CCOnto relevant to the example presented in this manuscript.

Top-Level Class	Level 1 Subclasses	Level 2 Subclasses
Person		
Character	Characteristic Adaptation	Appearance, Body Image, Goal, Motivation
	Mental Emotional Functioning	Appraisal Process, Psych. Response To Emotion Process, Appraisal, Affective Process
	Personality	Openness, Conscientiousness, Extraversion, Agreeableness, Neuroticism, Trait Anxiety, Behavioral Activation, Behavioral Inhibition, etc.
Behavior	Singular Behavior	Activity, Emotional Behavioral Process, Consumption Beh., Entertainment Beh., Sleep Beh., etc.
	Habitual Behavior	Chronotype, Food Diet, Sleep, Sleep Hygiene, Sleep Quality, Sleepiness, Exercise, etc.
	Learning Behavior	Learning Frequency, Timing, Medium, Modality, Quality, etc.
Situation	Task	Performed Task, Cognitive Task, Sleep Task, etc.
	Environment	
	Social Situation	
	Learning Situation	
Coordinate	Component	Arousal, Valence
	Radius	
Culture	Ethnic Identity	
	Religion	
	Nationality	
	Residence	
Food	Basic Food	
	Nutrient	
Well-Being	Emotional Well-Being	
	Physical Well-Being	
Disease	Sleep Disorder	
	Pathological Mental Response	

Character consists of three main subclass clusters, namely characteristic adaptation, mental emotional functioning, and personality. Characteristic adaptations can vary over time, such as appearance or goals. Mental emotional functioning includes all affect-related concepts incorporated from the emotions ontology (Smith and Ceusters, 2010 and Hastings et al., 2012) and readjusted to fit CCOnto. Personality consists of the five FFM traits, trait anxiety, and BIS/BAS traits (Pickering and Corr, 2008). Behavior is represented as scores of specific behavior-related tasks. We distinguish singular, habitual, and learning behavior and belonging tasks. The situation is thus represented as a specific task, environment, or social and learning situations. The coordinate represents continuous, categorical emotions and is reused from EmOCA (Berthelon and Sander, 2013), alongside the stimulus and impact (of a personality trait) classes.

### Use Case: Psychological Assessment

**FIGURE 4:**
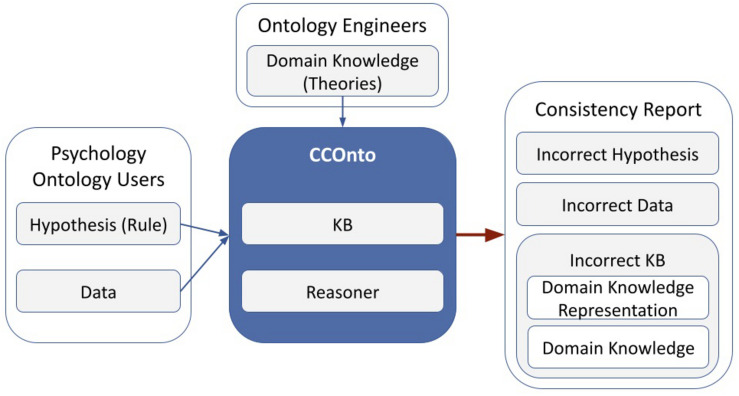
An overview of a psychologically driven usage scenario of the ontology-based theoretical framework.

As described under the sections “Methods” and “Aim of the Present Study,” in this manuscript, we apply the CCOnto ontology CCOnto to the specific question of behavior change among university students during COVID-19. Figure 4 gives an overview of the different usage scenarios of the CCOnto ontology. Notably, the added value and purpose of an ontology is to test the consistency of different hypotheses with respect to the knowledge representation model and the dataset. Inconsistencies indicate one of 4 options to be investigated by the knowledge engineers and researchers: (1) incorrect hypothesis, (2) incorrect or inconsistent data, (3) inconsistent representation of the underlying concepts with respect to the dataset, or (4) incorrect underlying theories. The latter would be of particular benefit when comparing two representation theories of different situation, state, or trait markers, e.g., two theories of emotions. Due to being as theory-agnostic as possible, the ontology supports comparing between different theories. This would help indicate whether some theories are more relevant in some cases as opposed to others.

To highlight the basic usage scenario presented in Figure 4, we used the ontology to test the initial hypotheses about mental and behavior change among university students conceptualized and described in Herbert et al. (2021a). The output of the ontology is aligned with the previously conducted statistical analysis in Herbert et al. (2021a). In this manuscript, we present how we evaluate the ontology usage in the context of categorically structuring information about COVID-19 related behavior change. As proof of concept, we rely on the concept and design of the already conducted psychological online survey (Herbert et al., 2021a) conducted during the first COVID-19 pandemic to assess behavior change across psychological domains among university students studying in Egypt or Germany. The survey items assess different psychological aspects of behavior related to teaching and learning, social behavior, mental health, and well-being [please see Herbert et al. (2021a)]. The results of this psychological study showed several interesting findings confirming that university students across cultures and countries have been seriously affected by the COVID-19 pandemic (Herbert et al., 2021a).

#### Data Structures

The online psychological survey used a mix of psychological measures, standardized psychological questionnaires, and open and closed survey items [please see Herbert et al. (2021a)]. The measures included (1) the big five personality traits (John et al., 1991), (2) depression measures screening for depressive symptoms (PHQ-2; Löwe et al., 2005), (3) state and trait anxiety markers (STAI, Watson et al. (1988), (4) feelings toward and during the pandemic, (5) worries about mental and physical health, (6) perceived difficulties in identifying and representing emotions (TAS-20, Bagby et al., 2014), (7) social distancing behavior and sentiments toward it, (8) learning behavior and sentiments toward it, (9) changes in behavioral patterns (sleep, eating, exercising), and (10) general emotions through the self-assessment manikin (SAM) (Bradley and Lang, 1994); for an overview see Herbert et al. (2021a).

## Implementation of Psychological Factors Involved in Student Education and Behavior During the Pandemic

Based on this psychological conception of behavior change and the given design to investigate it empirically through psychologically standardized, valid, and reliable conceptualization and operationalization in terms of psychologically valid assessment tools (Herbert et al., 2021a), we used the CCOnto to categorize the chosen research measures. This approach is not limited to the current use-case but can be applied to similar domains related to human behavior.

The following will show how the research methods and measures were aligned with the ontology to validate and test whether novel connections would arise from the process. This is done by mapping the input options to the ontology concepts through the relations between a person and the different concepts within the ontology. Thus, any input value will be mapped to its superclass (the concept it belongs to), leading to the categorization step. Table 2 gives an overview of the central relations used to realize this within CCOnto. It is important to note that this is a very abstracted notion of the ontology entities for illustrative purposes. Each of the relations in Table 2 has the person as domain and the different categorical factors as the range. The discussed psychological factors are thus each realized by the corresponding relation in Table 2.

**TABLE 2:** Overview of the main relations mapping between uncategorized input and hierarchical factors within the ontology.

Relation	Range (Upper-level Entity Mapping to Input)	Related Factors
hasDateOfBirth	Date	General
hasGender	Literal Value	General
has Marital Status	Literal Value	General
has Country of Origin	Country	General
has Country of Residence	Country	General
hasCulture	Culture	General
hasAffect	Coordinate (Valence, Arousal)	Character
hasAffect	Affective Process	Character
has Bodily Feeling	Bodily Feeling	Character
has Emotion Process	Emotion Process	Character
has Mood Process	Mood Process	Character
has Subj Emotional Feeling	Subjective Emotional Feeling	Character
has Appraisal	Appraisal	Character
has Physiological Response	Physiological Response To Emotion Process	Character
has Appraisal Process	Appraisal Process	Character
has Personality	Personality	Character
has Activity	Activity	Behavior
has EmotionalBehavioralProcess	Emotional Behavioral Process	Behavior
has Consumption Beh.	Consumption Beh.	Behavior
has Entertainment Beh.	Entertainment Beh.	Behavior
has Sleep Beh.	Sleep Beh.	Behavior
has Chronotype	Chronotype	Behavior
has Food Diet	Food Diet	Behavior
has Sleep	Sleep	Behavior
has Sleep Hygiene	Sleep Hygiene	Behavior
has Sleep Quality	Sleep Quality	Behavior
has Sleepiness	Sleepiness	Behavior
has Exercise	Exercise	Behavior
in Environment	Environment	Situation
hasTask	Task	Situation
in Social Situation	Social Situation	Situation
has Learning-Oriented Affect	Mental Emotional Functioning	Learning
has Learning-Related Behavior	Learning Behavior	Learning
in Learning Related Situation	Learning Situation	Learning

*The relations have the person as a domain and the categorical ontology factors as their range.*

### General Identifying Factors

Demographics are one primary identifier of human beings. Gender, age, marital status, countries of origin, and residence all shape and identify behavior. For example, a married university student would require different inference rules to determine procrastination. An international student has different representation requirements than a student visiting a university in his/her hometown, especially in the case of a lockdown.

A person’s socio-cultural embeddings dictate how they perceive certain things and, in turn, how they react toward them [e.g., Banks (1988) and Park (2002)]. We denote this as culture, which refers to ethnicity, religion, and nationality for modeling purposes. We do not differentiate between subtypes of culture but rather consider it as one concept. This means that a single individual can have many values for culture representing their ethnicity, religion, and nationality.

### Character: State and Trait Markers

#### Affective Experience

If we consider the categorization of emotional, mental functioning alone, we can see why utilizing an ontology is inherently suited for categorizing and capturing data. Table 3 gives an overview of the main classes and subclasses involved in mental emotional functioning, as incorporated from the Emotions Ontology, to help understand the below example.

**TABLE 3:** The main classes and subclasses involved in mental emotional functioning, as incorporated from the Emotions Ontology.

Top-Level Class	Level 1 Subclasses	Level 2 Subclasses	Level 3 Subclasses
Mental Emotional Functioning	Affective Process	Bodily Feeling	Hunger, Pain, Thirst, Physical Pleasure, etc.
		Emotion Process	Amusement, Anger, Anxiety, Boredom, Compassion, etc.
		Mood Process	Anxious Mood, Cheerful Mood, Gloomy Mood, etc.
		Subjective Emotional Feeling	Feeling Alert, At Ease, Bad, Calm, etc.
	Appraisal	Appraisal Of Being Dislikes, Being Liked, Avoidability Of Consequences, Loss, etc.	
	Physiological Response To Emotion Process	Becoming Pale, Blushing, Perspiring, Shivering, etc.	
	Appraisal Process		

*Only a sample of the included distinctive values is included for illustration.*

For instance, consider the input” I feel… bad, introverted, anxious, pain, and fat.” resulting from the free linguistic task (Herbert et al., 2021a) of describing own feelings in response to the pandemic included in the dataset described in Subsection “Use Case: Psychological Assessment.” This input data results from different state and trait markers and would require linguistic and human analysis to categorize. However, once we input it into the ontology, it will implicitly be divided to match the different belonging superclasses. Thus, the resulting alignment to ontology concepts would be that the current individual has (1) a “subjective emotional feeling” with the value bad, (2) a “perceived personality trait” with the value introvert, (3) a “mood process” with the value anxious, (4) a “bodily feeling” of pain, and (5) a “body image” (characteristic adaptation) of being fat. It is important to note that no causality relations are included in the data. Thus, the ontology would just serve as categorizing the set of current state and trait markers. Any intrinsic causalities might be suggested by the ontology relations and the chosen psychological theories included in the ontology. For example, a specific theory hypothesizes a causal relation between x and y. If this theory aligns with the current researcher’s hypotheses, it can be included as a set of presets in the ontology. However, if the current research advocates another (not necessarily aligning theory), the ontology user can remove these causalities from the ontology.

However, this is not only limited to categorizing free text description tasks but also to unifying answers from different question types and response scales. Accordingly, we can support representing an individual having discrete emotions and coordinate-based emotions resulting from list choice entries and the SAM scale (Bradley and Lang, 1994), respectively. This is achieved by having two hasAffect properties for a given human being; the first has the emotions class as an object, and the second has a coordinate (arousal and valence) class as an object. This provides the added advantage of enabling the cross-validation of the two reported emotions. Thus, it could help indicate certain biases in a person’s answers or highlight differences in emotion perception, especially if targeted toward two constructs, e.g., threat perception (SAM) and general current emotions (discrete emotions).

#### Personality

The same applies to identifying personality. Mapping data to the ontology enables aligning results from different personality models and questionnaires to common traits. Thus, instead of performing empirical experiments to test validity and mappings of different scales, they can be represented alongside each other as traits (subclasses of personality). Using ontology validity checking, we can compare whether the entries are compatible with each other or if an inconsistency arises from unifying the inputs of two personality models.

### Behavior

Behavior is defined as any “internally coordinated response(s) (actions or inactions) of humans (animals) (individuals or groups) to internal or external stimuli, via a mechanism that involves nervous system activity.” within the Gene Ontology (Ashburner et al., 2000; Carbon et al., 2021). We focus on a more abstracted notion of behavior that does not necessarily focus on the lower-level behavioral functions unless they directly serve to infer higher-level ones, such as physiological responses to emotions and affective representations related to threat perception during the pandemic.

All behavior-related relations in Table 2 can be used to represent information about a single occurrence of behavior, recurring habits, and behavior change with respect to the pandemic. Also, most of them can be regarded as stand-alone well-being-related behaviors. Thus, depending on each input instance, the information is mapped to one or many of the below indicators simultaneously.

#### Behavior Frequency

The main distinction we make when dealing with behaviors is differentiating between habitual and singular behaviors. This is important for easing the reasoning on the different behavior occurrences (as explained in more detail below). Singular behaviors occur once or non-frequently. They are considered stand-alone events. Habitual behaviors are those that repeat over a specific time or periodically in a specific pattern. Such behaviors need to be considered based on their patterns. We cannot treat both behavior types equally as each of them usually have different indications and thus need to abide by different rules.

For instance, imagine a student skipped the assignment on his schedule one day. This could have several explanations like being busy with other things on that day, not being in the mood, forgetting, etc., However, imagine the skipping behavior repeats over many days where the student always adds the assignment to the to-do list but fails to complete it. This procrastination behavior (Karatas, 2015) could indicate a more serious lack of motivation, for example. Another example is a bad night’s sleep versus a repeated pattern on sleepless nights, where the former can be explained by bad sleeping conditions or a passing nightmare, for example. At the same time, the latter indicates a more profound problem like insomnia. Thus, differentiating between behavior frequency and patterns is an integral categorization factor.

#### Behavior Change

Especially in a context like that of the COVID-19 pandemic, we cannot consider behavior as an absolute concept but rather as a relative one. We thus consider behavior changes and continuous behaviors. Behavior change refers to singular or habitual behavioral patterns that are different from previously consistent habitual behavior. A previous singular behavior cannot be considered when representing an instance of behavior change. However, one singular behavior that opposes an established habitual pattern is considered an instance of behavior change. To illustrate, imagine the same student with the assignment. If we add the extra fact that the student always finishes assignments as soon as they are announced and never postpones an item on the to-do list. This would change the implications of postponing the assignment even once, as this is a grave behavior change for the student.

The best indicator is thus combining behavior change considerations with mappings of singular and habitual behavior instances. However, it is not always the case that both pieces of information are simultaneously present, requiring the reasoning with only the available information.

#### Health Behavior and Well-Being

Well-being consists of the set of behaviors needed for a person to maintain a healthy lifestyle. There are six dimensions of well-being: emotional, occupational, physical, social, intellectual, and spiritual Hettler (1976). For the sake of brevity, in this manuscript, we will focus on emotional and physical well-being, as they will be used to highlight the alignment with the collected data.

Emotional well-being is represented using affective processes, appraisal, and physiological responses to emotion. Physical well-being is represented in CCOnto by its main building blocks: nutrition, physical activity, and sleep (see Tables 1, 2).

We will discuss the main classes representing physical well-being. The enumeration of the different foods and activities is integrated from the HeLiS ontology (Dragoni et al., 2018). To enable representing all needed physical well-being factors, we extend the ontology with the extra needed classes and relations shown in Tables 1, 2, e.g., sleep information, consumption behavior, exercise, etc., We also demonstrate the related evaluation rules, i.e., hypotheses embedded in the ontology to evaluate physical well-being components. The rules can always be set by the researcher using the ontology depending on the use-case. The rules are embedded into CCOnto using the Semantic Web Rules Language (SWRL), which has a format similar to if\then statements: A -> C. If the information in the antecedent A is matched, then the results in the consequent C (value binding within the ontology classes) are added.

1. Sleep is considered to be a vital predictor of well-being. Habitual behaviors (e.g., chronotype and sleep hygiene) and subjective experience (e.g., sleep quality and sleepiness) are integral aspects of sleep (Duggan et al., 2014). Table 4 contrasts sample instances of healthy and unhealthy sleep hygiene (Stepanski and Wyatt, 2003; Mastin et al., 2006; Irish et al., 2015). These instances are mapped to the ontology as rules for determining the sleep hygiene value from input data. An abstracted rule resulting from the first entry in Table 4 would have the following format:
  consume (?p,?LightFood) ^ has Activity(?p, ? moderate activity) - > has Sleep Hygiene(?p, high Sleep Hygiene).
  Personality has a significant association with sleep health. For example, Duggan et al. (2014) showed that low Conscientiousness and high Neuroticism were the best predictors of poor sleep. Thus, the ontology can be used to map between personality variables and sleep health variables, e.g.,
  Person (x?) ^ has Sleep(?x,?y) ^ PoorSleep(?y) ^ has Conscientiousness(?x,?z) - > Low Conscientiousness(?z).
2. Physical Activity is defined as any bodily movement produced by skeletal muscles requiring energ expenditure 1. Exercise is a planned, structured, repetitive physical activity aiming to improve or maintain one or more physical fitness (Caspersen et al., 1985). Physical activity can be an indicator of personality traits. There is evidence for negative correlations between neuroticism and physical activity and positive correlations between extraversion and physical activity (Rhodes, 2006).
3. Nutrition studies the effect of food and drinks on our bodies regarding the essential nutrients necessary to support human health. Good nutrition means obtaining the right amount of nutrients from healthy foods in the right combinations. A poor diet is a known risk factor for overweight, obesity, and chronic lifestyle diseases, including hypertension, type 2 diabetes, and coronary heart disease (World Health Organization, 2009). Models of environmental and socio-cultural influences show that socioeconomic location, social support, and personality are associated with dietary choices (Giskes et al., 2011; Lunn et al., 2014).

**TABLE 4:** Some of the behaviors that cause poor sleep hygiene and recommendations for good sleep hygiene.

Inadequate sleep hygiene	Adequate sleep hygiene
Alcohol, tobacco, or caffeine consumption in the period preceding bedtime	Hunger may disturb sleep, a light bedtime snack seems to help individuals to sleep
Daytime napping	Sleeping as much as needed to feel refreshed and healthy during the following day
Variable wake-up or bedtimes	Regular arousal time in the morning
Engaging in emotionally upsetting events too close to bedtime	Caffeine in the evening disturbs sleep
Non-sleep activities (e.g., television watching, reading, studying, snacking, etc.)	Avoiding Alcohol, Nicotine use before bedtime
Performing activities that require high levels of concentration shortly before bedtime	Sleeping in a quiet and comfortable environment
Mental activities	Regular Exercise

### Situation

Even with recent theories advocating situated-ness (Barsalou, 2019), embodiment (Niedenthal et al., 2005), and environmental psychology (Russell and Ward, 1982), the situation is not often included as a potent representative of behavior. As advocated by Character Computing (El Bolock, 2020), however, the situation is a central concept representing behavior and thus the third member of the CBS triad modeled by CCOnto. We define a situation as any external factors and conditions of an individual. The situation is to be represented by different (non-mutually exclusive) aspects.

#### Environment

When talking about a situation and how it affects behavior, one of the main things that come to mind is the surrounding environment, i.e., everything around us (Russell and Ward, 1982). The environment is defined as “the conditions that you live or work in and the way that they influence how you feel or how effectively you can work.” Fielding and Davis (1989) define the environment as surroundings, influence, and conditions acting on an organism. In Last (2001), the environment is defined as” all which is external to the human host. It can be divided into physical, biological, social, cultural, any or all of which can influence the health status of populations.” This refers to people, living entities, physical objects, and places surrounding an individual at any given time and in different settings. An environment also consists of the events a person is going through, e.g., the current COVID-19 pandemic. The culture of a person also makes up their environment.

#### Tasks

We represent the situation as specific tasks to be performed. Tasks can be literal or figurative. Literal tasks are, for example, performing a cognitive task or taking a test. Figurative tasks represent behaviors that are performed, like working out or sleeping. We consider tasks as situations because they are setups that an individual is in and that affect behavior.

#### Social Situation

An individual’s social situation is a significant determinant factor of behavior and thus a main representation point. The living situation is one aspect of the social situation. For example, whether an individual is locked down far away from home or with one’s family highly affects and identifies behavior. The change in the living situation is also essential, e.g., students usually living in dorms but having to move back home due to the lockdown situation. Depending on other factors like the individual’s trait markers, this experience would improve or worsen state markers and resulting behaviors like learning and well-being. Material factors like internet connectivity and the availability of needed hardware to perform different tasks (learning or leisure-oriented) are other determinants of states and behaviors. Social interactions, which the pandemic has severely compromised, are crucial situation markers. The social distancing behaviors map to the social situation an individual is in. Whether friends and surrounding people are adhering to the same measures is a situation marker that highly affects state markers and behaviors.

### Learning

Of particular importance to the purposes of this paper are the different learning patterns and behaviors. Learning factors can be interpreted as and are composed of entities belonging to different categories. Representing the variables related to learning and educational behavior thus benefit the most from the ontology-based categorization.

#### Affective Representations Related to Education

We consider perceived emotions toward learning, which is a state marker component. This includes feelings toward learning and its different modalities in general and during the pandemic. Online learning and university work are sample concepts that each student can emotionally evaluate. By considering these subjective sentiments and their perceived change before and after the pandemic, we include the new notion of emotion change, similar to behavior change considerations. Here, it becomes clear that we need the ontology-provided relative representation of emotional states.

#### Behavior Aspects of Learning

Learning can also be represented and thus categorized as behaviors. Different students have different learning habits, patterns, and methods. Some regularly keep up with the study material, while others catch up at the last minute. Some students prefer to study alone while others can only digest information in a group. Most importantly, some students thrive in self-learning while others are lost without the semblance or order provided by live teaching. It is, thus, necessary to represent the learning behaviors of an individual. This, in turn, requires representing learning behavior changes and supporting the notion of relative learning behaviors alongside absolute ones. Learning patterns need to be represented before and after the pandemic-driven teaching modality change.

#### Situation Aspects of Learning

Finally, learning is also a situation in itself and needs to be represented and categorized as such. Here, we distinguish between two different situation representations of learning.

1. Learning is a single task or a collection of tasks to be performed (given the definition of a task presented in section “Tasks”). Learning-related tasks can be divided into different well-defined explicit tasks like assignments, tests, and projects. Learning can also be represented as abstract tasks of knowledge acquisition like understanding a subject matter or covering a course’s learning unit. Both representations are needed for providing different insights. For example, a student’s conscientiousness can be categorized by the former representation, while their focus can be identified and measured by the latter.
2. Learning is also represented as a situation from a different aspect. Learning is associated with a specific environment, which is the university itself. Learning from home instead of at the university is a change in the student’s environment (another representation aspect of a situation). Representing this situation change and relative environments are required to adequately represent the situational aspects of learning enabled by the ontology model.

### Discussion: Psychoeducational Implications of Psychologically Driven Ontologies

The proposed approach of categorizing data (specifically psychoeducational behavior data) using ontologies has many implications for the domain of psycho-education building on the outlined use-case. Using the ontology representations, we showed how learning is simultaneously represented by state markers, behaviors, and situation factors. This representation mode sheds new insights into understanding learning from a holistic psychological perspective. Learning behavior depends not only on educational factors or learner’s personality but also on learner states, situation, and other behavior. Given that psychoeducational data is currently often multimodal or implicitly included in other data sources using ontologies similar to CCOnto are integral to consolidating results through knowledge-based representation as outlined above.

Two notable implications of the proposed ontology are enabling, (1) knowledge inference and dataset completion in relation to human factors and (2) combining the high predictive power of machine and deep learning with the high interpretative power of ontologies.

#### Knowledge Inference and Dataset Completion

As demonstrated in the previous section, we can see how our ontology and its entities can categorize unstructured data. The ontology-enforced data categories can be mapped to the manually labeled data categories presented in Herbert et al. (2021b). For instance, the state and trait markers map to affective experiences, personality, and self-concept. Let’s consider the different aspects of learning represented in section “Learning.” We will see that learning factors cannot be all categorized as such and that learning-related variables are a composite of different factors. The same holds for data resulting from the same input measure, which can be categorized into different markers and behavior domains (see the example of the free text description tasks from section “Character: State and Trait Markers”).

The inference engine embedded within the ontology can be used to infer hierarchy specifications. For example, without specifying these facts, the reasoner can infer that a learning instance has the dual representation of a task and behavior. Not all existing relations need to be explicitly defined within the ontology. By leveraging the ontology-enforced entity hierarchy, we can infer that relations that hold for concepts also hold for their sub-concepts. For example, by defining that a person “hasTrait” of the type personality traits, we intrinsically know that a person can have trait values for extroversion, anxiety, and behavioral inhibition, for instance (if these are the traits of interest for the current consideration).

Another use of inference is predicting values that are missing from the current ontology. For example, suppose the collected data has not insights about the behavioral inhibition trait. In that case, we can embed rules for relating behavioral inhibition with existing data entries, e.g., extraversion and trait anxiety. The rules can be chosen and adapted as seen fit, and the resulting inferred values would adapt according to the rule change. For instance, if we consider the results of the study presented in Vreeke and Muris (2012) for the rules, we will define that (1) extraversion is a strong negative correlate of behavioral inhibition regardless of anxiety and (2) anxiety and behavioral inhibition are strongly correlated. Thus, these rules would help the reasoner infer behavioral inhibition categorization (qualitative, not quantitative) of children given their anxiety and extraversion traits. This can be applied to different rules and specified markers and entries to be inferred.

One main advantage of these knowledge inferences is dataset completion (Liu et al., 2017; Schneider and Šimkus, 2020).

Finally, the same automated reasoning approaches can be applied to predicting specific markers or behaviors of new individuals’ interest (data entries). The modeling of the concepts involved in psychoeducational variables enables having a conceptual structure to be used as a basis for multiple use-cases. One such use-case is raising the awareness of educators and family members to perceived threat factors that might be affecting the student’s emotional well-being or educations performance.

#### Combining Ontology Modeling With Machine Learning

Artificial intelligence and machine learning are among the most frequently used techniques for data and behavior prediction. However, their performance is contingent not only on the availability of data but also on the input data quality. This is referred to as “garbage in and garbage out,” i.e., the output of any algorithm, no matter how complex, is only as good as the processing of the input data (García et al., 2016). We require structured information to allow machines to learn and predict human behavior. Thus, transforming unstructured data to structured information is central to all technology-based solutions, especially human-centric ones. Information systems often rely on computational ontologies are often used for aligning heterogeneous and completing insufficient data (Liu et al., 2017; Schneider and Šimkus, 2020).

Machine learning algorithms are often used to make predictions about different factors based on existing data. Previously, semantic modeling (ontologies) and machine learning have been considered two different approaches that cannot be unified. However, in recent times with the increase of data amounts and complexity, it had become apparent that the most probable way forward is in combining both fields and leveraging the one for the other. Following the approach proposed in this paper, we can structure and process data through ontologies for machine learning and use ontology-based reasoning to explain black-box machine learning models. In Kulmanov et al. (2020), a detailed review of using ontologies with machine learning and semantic similarities within bioinformatics is presented.

In Herbert et al. (2021a), we had already investigated predicting anxiety and negative emotions via machine learning models with an average accuracy of 70%. However, it became apparent that a detailed explanation of the reasons behind such predictions and the couples correlating factors was of high importance to psychology research. Thus, combining the previous ML models with the current proposed ontology categorization approach would greatly benefit for furthering behavior prediction targeted toward mitigation. It would also provide information about the contributing factors.

## Conclusion

We presented a theoretical ontology-based framework for modeling different aspects of behavior in relation to psychological state and trait markers. We highlighted how a computing ontology representing behavior and its related factors could help automatically organize information out of unstructured data through identifying and categorizing concepts. We presented this by demonstrating it on an example of categorizing the markers related to student behavior and its different aspects during the first COVID-19 lockdown as conceptualized and provided in Herbert et al. (2021a) and as summarized in this manuscript. Using the ontology, we categorized the state and trait markers related to different aspects of student behavior, including learning, worrying, health, and social. The ontology also categorizes the behaviors and situation identifiers. The ontology enables psychology researchers to test specific hypotheses against datasets and in lab experiments.

We also discussed the potential extensions of the same theoretical framework to further understand the interactions among the different variables during the pandemic.

As outlined in Subsection “Discussion: Psychoeducational Implications of Psychologically Driven Ontologies,” in the future, the proposed ontology can be extended to cover further aspects of behavior for different purposes. For example, it can be extended to target mental well-being. It can also be applied to domains other than education and different samples, e.g., extending it to different age groups other than students. The proposed framework can be extended to the scope of and applied to any behavior-related dataset. The summary of benefits and usage scenarios of the ontology-based framework include:

1. Hypothesis validation against dataset through rules.
2. Missing input prediction through reasoning.
3. Dataset processing and organization.
4. Traceable, explainable predictions.

In the long run, a more extended application of the framework can enable testing whole psychological theories (as a set of hypotheses or as part of the knowledge base).

## Data Availability Statement

There is no data to share. The structure of the online psychological survey we refer to in the ontology can be found in Herbert et al. (2021a) under revision and is explained in detail in the text of this manuscript. No data from the empirical study is used, but the concept and design of the study were used and implemented in the ontology.

## Author Contributions

AB and CH: conceptualization, theory, and writing – review and editing. AB: formal analysis – ontology. AB, CH, and SA: validation. AB: writing – original draft. CH and SA: revision of the draft for critical intellectual input, supervision, and funding. CH: project administration. All authors approved the final version of the manuscript to be published.

## Conflict of Interest

The authors declare that the research was conducted in the absence of any commercial or financial relationships that could be construed as a potential conflict of interest.

## Footnotes

1 https://github.com/CharacterComputingResearch/CCOnto
